# Draft genome of *Paraburkholderia caballeronis* TNe-841^T^, a free-living, nitrogen-fixing, tomato plant-associated bacterium

**DOI:** 10.1186/s40793-017-0294-7

**Published:** 2017-12-16

**Authors:** Fernando Uriel Rojas-Rojas, Erika Yanet Tapia-García, Maskit Maymon, Ethan Humm, Marcel Huntemann, Alicia Clum, Manoj Pillay, Krishnaveni Palaniappan, Neha Varghese, Natalia Mikhailova, Dimitrios Stamatis, T. B. K. Reddy, Victor Markowitz, Natalia Ivanova, Nikos Kyrpides, Tanja Woyke, Nicole Shapiro, Ann M. Hirsch, Paulina Estrada-de los Santos

**Affiliations:** 1 Instituto Politécnico Nacional, Escuela Nacional de Ciencias Biológicas, Prol. Carpio y Plan de Ayala s/n. Col. Santo Tomás Del. Miguel Hidalgo, C.P. 11340 Mexico City, Mexico; 2 Department of Molecular Cell and Developmental Biology, Los Angeles, CA 90095 USA; 3 DOE Joint Genome Institute, 2800 Mitchell Drive, Walnut Creek, CA 94598 USA; 4 Molecular Biology Institute, University of California-Los Angeles, California, Los Angeles 90095 USA

**Keywords:** Paraburkholderia *caballeronis*, Tomato plant, Rhizosphere, Nitrogen fixation, Root nodulation

## Abstract

10.1601/nm.26956 *caballeronis* is a plant-associated bacterium. Strain TNe-841^T^ was isolated from the rhizosphere of tomato (*Solanum lycopersicum* L. var. lycopersicum) growing in Nepantla Mexico State. Initially this bacterium was found to effectively nodulate *Phaseolus vulgaris* L. However, from an analysis of the genome of strain TNe-841^T^ and from repeat inoculation experiments, we found that this strain did not nodulate bean and also lacked nodulation genes, suggesting that the genes were lost. The genome consists of 7,115,141 bp with a G + C content of 67.01%. The sequence includes 6251 protein-coding genes and 87 RNA genes.

## Introduction

10.1601/nm.26956 *caballeronis* was isolated in the State of Mexico, Mexico from the tomato rhizosphere as a free-living, nitrogen-fixing bacterial species [1]. It was described as 10.1601/nm.25163 and found to nodulate *Phaseolus vulgaris* L. [2]. Most nodulating bacteria are isolated from root nodules but this was not the case for 10.1601/nm.25163, which was isolated from rhizospheric soil. Given the ability of this bacterium to fix nitrogen under both free-living and symbiotic conditions, this type strain was selected for genome sequencing to study its nitrogen-fixing and other plant-growth promoting activities. However, after analyzing the genome, we found that the genes for fixing nitrogen were present but nodulation genes were not. We carried out several unsuccessful tests to check the ability of this strain to nodulate *P. vulgaris*, strongly suggesting that the strain had lost the *nod* genes. The genome sequence of 10.1601/nm.26962 TNe-841^T^ was obtained in cooperation with JGI-DOE. The type species is TNe-841^T^ (= 10.1601/strainfinder?urlappend=%3Fid%3DLMG+26416 ^T^ = 10.1601/strainfinder?urlappend=%3Fid%3DCIP+110324 ^T^).

## Organism information

### Classification and features

**Fig. 1:**
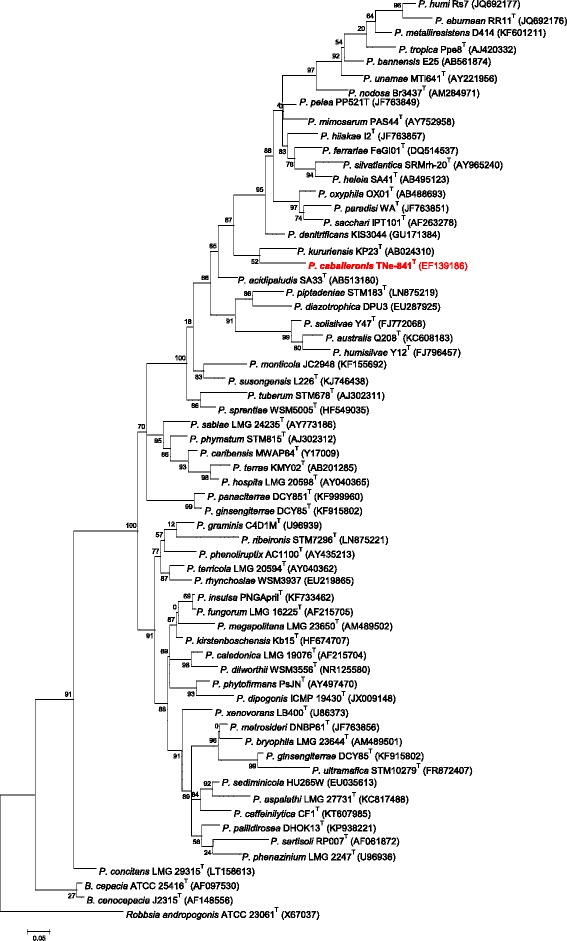
Phylogenetic tree highlighting the position of *Paraburkholderia caballeronis* TNe-841^T^ in relation to other *Paraburkholderia* species. *Burkholderia* and *Robbsia* were used as outgroups. The bar represents the number of expected substitutions per site under the GTR + G model. The sequenced strain is indicated in red

**Fig. 2:**
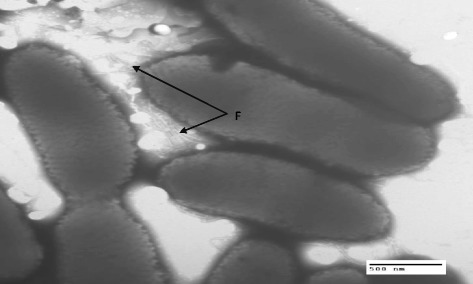
Transmission Electron Microscopy negative stain of *Paraburkholderia caballeronis* TNe-841^T^. The strain was grown on LB medium and a loop-full of cells was gently suspended in 1 mL distilled water. A drop of the suspension was placed on a formvar-coated copper grid and air-dried for 20 min to allow the cells to adhere. The grid was then covered for 20 s with a solution of 0.5% uranyl acetate, the excess liquid was removed with a filter paper, and then air-dried. A JEOL JEM-1010 transmission electron microscope, operated at 60 kV, was used to observe and photograph negatively stained preparations. F, stands for flagella

10.1601/nm.25163 TNe-841^T^ has been proposed to belong to the newly described genus *Paraburkholderia*. The last years, *Burkholderia* sensu lato has been subjected to some taxonomical changes, where the genus has been split to *Burkholderia, Paraburkholderia, Caballeronia* and *Robbsia andropogonis* [3–5]. However, this division has caused some skepticism, which has been expressed by The International Committee on Systematics of Prokaryotes, through the Subcommittee for the Taxonomy of *Rhizobium* and *Agrobacterium* discussed during the 12th Nitrogen Fixation Conference held in Budapest, Hungary on 25 August 2016 [6]. The Subcommittee stated: “Research efforts directed towards robust characterization and taxonomy of *Burkholderia* sensu lato species can help in realizing this agricultural potential. Clearly, large-scale phylogenomic study is required for resolving these taxa”. In order to analyze this issue and to provide generic limits in *Burkholderia* sensu lato, a large phylogenomic analysis was carried out using the amino acid and nucleotide sequence of 106 conserved proteins from 92 species [7]. The analysis performed with maximum likelihood unambiguously supported five different lineages: *Burkholderia* sensu stricto, *Paraburkholderia*, *Caballeronia*, *Robbsia andropogonis* and *B. rhizoxini*ca. To check the position of 10.1601/nm.25163 within 10.1601/nm.26956, the 16S rRNA gene sequence (ca. 1500 bp) was amplified and sequenced at Macrogen [8] with the universal primers fD1/rD1 [9]. The nucleotide sequence (accession number EF139186) was compared to other 10.1601/nm.26956 species using Muscle 3.57 for alignment [10]. A phylogenetic analysis was performed with ML using the PhyML program [11]. Among-site rate variation was modeled by a gamma distribution with four rate categories [12] with each category being represented by its mean under the GTR + G model. Tree searches were initiated from a BioNJ seed tree retaining the best tree among those found with NNI (Nearest Neighbor Interchange). The robustness of the ML topologies was evaluated using a Shimodaira-Hasegawa (SH)-like test [13]. The ML tree was obtained with the program MEGA version 5 [14]. The position of 10.1601/nm.26962 in the ML tree shows that it is close to *P. kururiensis* (Fig. 1). The colony morphology on BSE medium was uniform, 1 mm diameter, with entire margins that were convex, whitish, and translucent transparent. The cells are strictly aerobic Gram-negative, non-spore forming rod (0.49–0.69 μm × 1.2–2.7 μm) and have flagella (Fig. 2). Other phenotypic traits for this strain have been published before [2]. The strain has the following enzymes: arginine dihydrolase, urease catalase, and nitrogenase and associated proteins. It is also able to assimilate D-glucose, DL-arabinose, D-mannose, D-mannitol, N-acetyl glucosamine, gluconate, capric acid, malate acetate, D-ribose, D-xylose, D-adonitol, D-galactose, D-fructose, L-rhamnose, inositol, D-sorbitol, D-cellobiose, D-turanose, D-xylose, D-fucose, D-arabitol, potassium 2-ketogluconate, and potassium 5-ketogluconate (Table 1). Oxidase activity was weak. The strain grew on MacConkey agar plates at 29 °C and 37 °C, but weakly at 42 °C. 10.1601/nm.26962 TNe-841^T^ grew on LB and BSE agar plates at 15, 29, 37, and 42 °C and on LB plates at 29 °C with up to 5.0% NaCl.

**Table 1:** Classification and general features of Paraburkholderia caballeronis strain TNe-841 T [26]

MIGS ID	Property	Term	Evidence code^a^
	Classification	Domain Bacteria	TAS [27]
		Phylum *Proteobacteria*	TAS [28]
		Class *Betaproteobacteria*	TAS [29]
		Order *Burkholderiales*	TAS [30]
		Family *Burkholderiaceae*	TAS [31]
		Genus *Paraburkholderia*	TAS [32]
		Species *Paraburkholderia caballeronis* Type strain: TNe-841^T^ (LMG 26416 = CIP 110324)	TAS [2]
	Gram stain	Negative	TAS [2]
	Cell shape	Cells are single coccoids or in pairs	TAS [2]
	Motility	Motile	TAS [2]
	Sporulation	Non-spore forming	TAS [2]
	Temperature range	15-42 °C	TAS [2]
	Optimum temperature	30 °C	TAS [2]
	pH range; Optimum	6-7; 6	IDA
	Carbon source	D-glucose, DL-arabinose, D-mannose, D-mannitol, N-acetyl glucosamine, gluconate, capric acid, malate, acetate, D-ribose, D-xylose, D-adonitol, D-galactose, D-fructose, L-rhamnos, inositol, D-sorbitol, D-cellobiose, D-turanose, D-xylose, D-fucose, D-arabitol, potassium 2-ketogluconate, and potassium 5-ketogluconate	TAS [2]
MIGS-6	Habitat	Tomato rhizosphere soil	TAS [2]
MIGS-6.3	Salinity	Up to 5.0% NaCl (*w*/*v*)	TAS [2]
MIGS-22	Oxygen requirement	Aerobic	TAS [1, 2]
MIGS-15	Biotic relationship	Free-living	TAS [1, 2]
MIGS-14	Pathogenicity	Non-pathogen	NAS
MIGS-4	Geographic location	Mexico/Estado de México	TAS [1]
MIGS-5	Sample collection	2006	TAS [1]
MIGS-4.1	Latitude	18°59′11.7” N (18.986589)	NAS
MIGS-4.2	Longitude	98°50′44.0” W (−98.845552)	NAS
MIGS-4.4	Altitude	2010 m	NAS

^a^ Evidence codes - *IDA* Inferred from Direct Assay, *TAS* Traceable Author Statement (i.e. a direct report exists in the literature), *NAS* Non-traceable Author Statement (i.e. not directly observed for the living isolated sample but based on a generally accepted property for the species or anecdotal evidence). These evidence codes are from the Gene Ontology project [33]

#### Chemotaxonomic data

The following fatty acids were detected in strain TNe-841^T^ [2]: C14:0 (4.46%), C16:0 (21.77%), C16:0 2OH (2.3%), C16:0 3OH (6.2%), C16:1 2OH (3.81%), C17:0 cyclo (12.43%), C18:1 2OH (1.5%), C18:1 ω 7c (16.62%), C19:0 cyclo ω 8c (14.89%), summed feature 2 (5.9%), and summed feature 3 (8.3%). Summed feature two corresponds to C14:0 3OH and/or 16:1 ISO I, an unidentified fatty acid with equivalent chain length value of 10.928 12:0 ALDE or any combination of these fatty acids. Summed feature three corresponds to C16:1 w7c and/or C15:0 ISO 2OH.

## Genome sequencing information

### Genome project history

10.1601/nm.26962 TNe-841^T^ was sequenced at the JGI-DOE as a part of the project “Root nodule microbial communities of legume samples collected from USA, Mexico and Botswana” directed by Dr. Ann M. Hirsch. The goal of this project was to identify the microbial community housed within nodules of native legumes living in three arid or semi-arid, nutrient-poor environments in Mexico, Botswana, and the United States. Both 10.1601/nm.26956 and 10.1601/nm.1279 bacteria had been previously isolated from Mexico. 10.1601/nm.26962 TNe-841^T^ was chosen as the reference strain for a study of bacteria associated with native legume soils and nodules.

**Fig. 3:**
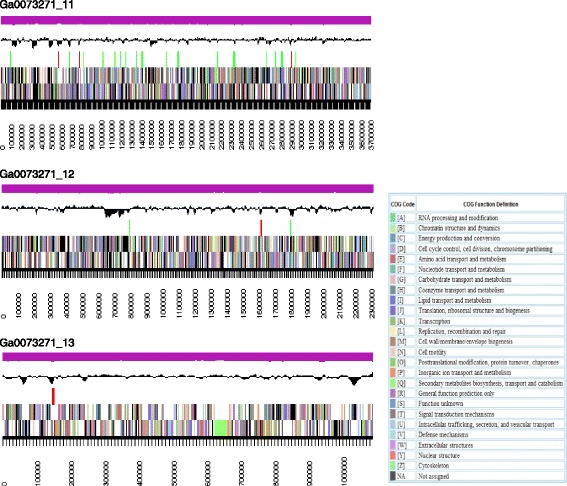
Graphical map of the 3 scaffolds of the genome of *Paraburkholderia caballeronis* TNe-841^T^. From the bottom to the top of each scaffold: Genes on forward strand (color by COG categories as denoted by the IMG platform). Genes on reverse strand (color by COG categories). RNA genes (tRNAs green, sRNAs red, other black). GC content, GC Skew

The complete sequence was finished on May 2015 and some features are presented in Table 2 and Fig. 3.

**Table 2:** Project information

MIGS ID	Property	Term
MIGS 31	Finishing quality	Level 3: Improved-High-Quality-Draft
MIGS-28	Libraries used	PacBio SMRTbell™
MIGS 29	Sequencing platforms	PacBio RS PacBio RS II
MIGS 31.2	Fold coverage	62.2X
MIGS 30	Assemblers	HGAP version 2.3.0_p5
MIGS 32	Gene calling method	Prodigal
	Locus Tag	BDK44
	GenBank ID	PRJEB16390
	GenBank Date of Release	October 20th 2016
	GOLD ID	Gp115207
	BIOPROJECT	PRJNA332775
MIGS 13	Source Material Identifier	LMG 26416^T^ = CIP 110324^T^
	Project relevance	Environmental

### Growth conditions and genomic DNA preparation

10.1601/nm.26962 TNe-841^T^ cells were grown in 5 ml of LB minus NaCl at 30 °C for 18 h at 120 rpm. The DNA extraction was done using Invitrogen’s Purelink™ Genomic DNA Mini Kit. The purified DNA was monitored for integrity by gel electrophoresis, and then sent to the JGI for sequencing.

Two surface-sterilized and rinsed seeds of *Phaseolus vulgaris* L. c.v. Negro Chapingo were planted per pot in surface-sterilized black pots (29.5 cm tall; 17 cm diameter) filled with autoclaved vermiculite:perlite (2:1) and watered with autoclaved 1/4 strength Hoagland’s –N medium. Two separate experiments were performed. The pots were either left uninoculated (sterilized water or Hoagland’s –N medium was added), inoculated with 10 ml of 10.1601/nm.26962 TNe-841^T^ diluted to OD_600_ = 0.2 or with 10.1601/nm.1651 DUS833, which was a positive control. Some pots were also watered with 1/4 strength Hoagland’s + N medium as an additional positive control. The appropriate medium was added twice weekly and the plants grown in a Conviron growth chamber under 16 h days/8 h nights at 24 °C.

### Genome sequencing and assembly

The draft genome of 10.1601/nm.26962 was generated using the PacBio sequencing technology [15]. A Pacbio SMRTbell™ library was constructed and sequenced on the PacBio RS platform, which generated 194,884 filtered sub-reads totaling 879.3 Mbp. All general aspects of library construction and sequencing performed at the JGI can be found at [16]. The raw reads were assembled using HGAP (version: 2.3.0 p5 protocol version = 2.3.0 method = RS HGAP Assembly.3 smrtpipe.py v1.87.139483) [17]. The final draft assembly contained 3 contigs in 3 scaffolds totaling 7.115 Mbp in size. The input read coverage was 62.2X.

### Genome annotation

Genes were identified using Prodigal [18] followed by a round of manual curation using GenePRIMP [19] for finished genomes and draft genomes in fewer than 10 scaffolds. The predicted CDSs were translated and used to search the NCBI nonredundant database, UniProt, TIGRFam, Pfam, KEGG, COG, and InterPro databases. The tRNAScanSE tool [20] was used to find tRNA genes whereas ribosomal RNA genes were found by searches against models of the ribosomal RNA genes built from SILVA [19]. Other non–coding RNAs such as the RNA components of the protein secretion complex and the RNase P were identified by searching the genome for the corresponding Rfam profiles using INFERNAL [20]. Additional gene prediction analysis and manual functional annotation was performed within the Integrated Microbial Genomes platform [21] developed by the JGI Walnut Creek CA USA [21].

The genome was also manually annotated at IPN and UCLA using the IMG platform [21].

## Genome properties

The final draft assembly of 10.1601/nm.26962 TNe-841^T^ contained 3 contigs in 3 scaffolds accumulating 7,115,141 bp in size (Table 3). The G + C content of the genome was 67.01%, which is very close to the one determined during the description of the species (66.0%) [2]. The genome was predicted to encode 6338 genes including 6251 protein-coding genes and 87 RNA genes (15 rRNAs 60 tRNAs and 12 ncRNA). The number of genes associated with general COG functional categories is shown in Table 4, in addition to other functions such as extracellular structures and mobilome.

**Table 3:** Genome statistics

Attribute	Value	% of Total
Genome size (bp)	7,115,141	100.00
DNA coding (bp)	6,194,680	87.06
DNA G + C (bp)	4,767,529	67.01
DNA scaffolds	3	100.00
Total genes	6338	100.00
Protein coding genes	6251	98.63
RNA genes	87	98.63
Pseudo genes	123	1.94
Genes in internal clusters	515	8.13
Genes with function prediction	5088	80.28
Genes assigned to COGs	4633	73.10
Genes with Pfam domains	5352	84.44
Genes with signal peptides	585	9.23
Genes with transmembrane helices	1456	22.97
CRISPR repeats	NF	

*NF* not found

**Table 4:** Number of genes associated with general COG functional categories

Code	Value	%age	Description
J	226	4.25	Translation ribosomal structure and biogenesis
A	1	0.02	RNA processing and modification
K	492	9.25	Transcription
L	124	2.33	Replication recombination and repair
B	1	0.02	Chromatin structure and dynamics
D	34	0.64	Cell cycle control Cell division chromosome partitioning
V	98	1.84	Defense mechanisms
T	274	5.15	Signal transduction mechanisms
M	361	6.79	Cell wall/membrane biogenesis
N	132	2.48	Cell motility
U	116	2.18	Intracellular trafficking and secretion
O	180	3.39	Posttranslational modification protein turnover chaperones
C	376	7.07	Energy production and conversion
G	367	6.9	Carbohydrate transport and metabolism
E	520	9.78	Amino acid transport and metabolism
F	102	1.92	Nucleotide transport and metabolism
H	285	5.36	Coenzyme transport and metabolism
I	300	5.64	Lipid transport and metabolism
P	338	6.36	Inorganic ion transport and metabolism
Q	190	3.57	Secondary metabolites biosynthesis transport and catabolism
R	514	9.67	General function prediction only
S	213	4.01	Function unknown
	1705	26.9	Not in COGs

The total is based on the total number of protein coding genes in the genome

## Insights from the genome sequence

10.1601/nm.26962 was originally described as a free-living, nitrogen-fixing bacteria with the ability to form nodules on *Phaseolus vulgaris* L*.* roots [2]. Although nitrogen fixation genes are present, nodulation genes were not found in the sequenced genome. Moreover, after the initial experiments, *P. vulgaris* nodulation was no longer detected in greenhouse bioassays in two different laboratories. This nodulation instability seems to be more frequent than originally assumed because a similar loss of nodulation ability has been reported with other 10.1601/nm.1619 strains isolated from nodules. The strains CCGE1002 and CCGE1003 (Marco Antonio Rogel CCG-UNAM, pers. comm.) also lost the ability to nodulate, but strain CCGE1002, which retains the ability to nodulate, was recovered from a stored sample. Its symbiotic plasmid was subsequently sequenced (NCBI BioSample PRJNA37719). In contrast, nodulation genes were no longer detected in the genome of strain CCGE1003 (NCBI BioSample PRJNA37721). A similar loss of nodulation genes was reported for two 10.1601/nm.1619 strains isolated from *Kennedia coccinea* [22] and *Gastrolobium capitatum* [23] in Australia.

Strain TNe-841^T^ also contains genes for degrading a large number of xenobiotics including aminobenzoate, atrazine, benzoate, bisphenol, caprolactam, chloroalkane, chloroalkene, chlorohexane, chlorobenzene, dioxin, ethylbenzene, fluorobenzoate, naphthalene, nitrotoluene, polycyclic aromatic hydrocarbons, styrene, toluene, and xylene.

ANI calculation was used to compare the genome of 10.1601/nm.26962 TNe-841^T^ and other 10.1601/nm.26956 species (Table 5). The ANI results showed that strains TNe-851^T^ correspond to a different species since the highest ANI value was 83.32. The accepted ANI cut-off for species is 95-96%, which corresponds to a DNA-DNA hybridization of 70% [24, 25].

**Table 5:** Average nucleotide identity of strain TNe-841^T^ with *Paraburkholderia* species genome

*Paraburkholderia* species	Average Nucleotide Identity (%)
*P. acidipaludis* NBRC 101816 ^T^	83.32
*P. ferrariae* NBRC 106233 ^T^	83.22
*P. tropica* LMG 22274 ^T^	83.05
*P. unamae* MTl-641 ^T^	82.96
*P. mimosarum* LMG 23256 ^T^	82.77
*P. silvatlantica* SRMrh-20 ^T^	82.77
*P. heleia* NBRC 101817 ^T^	82.68
*P. nodosa* DSM 21604 ^T^	82.68
*P. oxyphila* NBRC 105797 ^T^	82.64
*P. sacchari* LMG 19450 ^T^	82.59
*P. mimosarum* STM3621 ^T^	82.58
*P. eburnea* LMG 29537 ^T^	82.36
*P. bannensis* NBRC 103871 ^T^	82.31
*P. kururiensis* JCM 10599 ^T^	81.96
*P. sartisoli* LMG 24000 ^T^	81.82
*P. susongensis* LMG 29450 ^T^	81.82
*P. tuberum* STM678 ^T^	81.62
*Robbsia andropogonis* Ba3549	73.75

## Conclusions

10.1601/nm.26962 TNe-81^T^, is a plant-associated bacteria species with the ability to fix nitrogen, although the ability to nodulate legumes as shown in the original description was apparently lost. This nodulation instability seems to be rather common among nodulating bacteria, particularly 10.1601/nm.1619/10.1601/nm.26956. Our interest in studying the genome of 10.1601/nm.26962 TNe-841^T^ started when we found that this bacterium, isolated from the tomato rhizosphere, was able to nodulate bean. This led us to find out the identity of the original host for this species. Our work team has recently isolated a 10.1601/nm.26962 strain from bean nodules used as a trap with soil from an area where Mimosoideae plants are present (unpublished results). We are characterizing additional isolates from Mimosoideae plant nodules to try to establish if this plant might be the host of 10.1601/nm.26962 TNe-841^T^.

## Abbreviations

ANI: Average nucleotide identity
DOE: Department of energy
IMG: Integrated microbial genomes
JGI: Joint Genome Institute
ML: Maximum likelihood
NCBI: National center for biotechnology information
Pacbio: Pacific biosciences

## References

[1] Caballero-Mellado J, Onofre-Lemus J, Estrada-De Los Santos P, Martínez-Aguilar L (2007). The tomato rhizosphere, an environment rich in nitrogen-fixing *Burkholderia* species with capabilities of interest for agriculture and bioremediation. Appl Environ Microbiol.

[2] Martínez-Aguilar L, Salazar-Salazar C, Méndez RD, Caballero-Mellado J, Hirsch AM, Vásquez-Murrieta MS (2013). Estrada-de Los Santos P. *Burkholderia caballeronis* sp. nov., a nitrogen fixing species isolated from tomato (*Lycopersicon esculentum*) with the ability to effectively nodulate *Phaseolus vulgaris*. Anton Leeuw Int J G.

[3] Sawana A, Adeolu M, Gupta RS (2014). Molecular signatures and phylogenomic analysis of the genus *Burkholderia*: proposal for division fo this genus into the emended genus Burkholderia containing pathogenic organisms and new genus *Paraburkholderia* gen. Nov. harboring environmental species. Front Genet.

[4] Dobritsa AP, Samadpour M (2016). Transfer of eleven *Burkholderia* species to the genus *Paraburkholderia* and proposal of *Caballeronia* gen. Nov., a new genus to accommodate twelve species of *Burkholderia* and *Paraburkholderia*. Int J Syst Evol Microbiol.

[5] Lopes-Santos L, Castro DBA, Ferreira-Tonin M, Corrêa DBA, Weir BS, Park D, Ottoboni LMM, Neto JR, Destéfano SAL (2017). Reassessment of the taxonomic position of *Burkholderia andropogonis* and description of *Robbsia andropogonis* gen. Nov., comb. nov. Anton Leeuw Int J G.

[6] de Lajudie PM, Young JPW (2017). International committee on systematics of prokaryotes subcommittee for the taxonomy of rhizobium and agrobacterium minutes of the meeting, budapest, 25 august 2016. Int J Syst Evol Microbiol.

[7] Beukes C, Palmer M, Manyaka P, Chan WY, Avontuur J, van Zyl E, Huntemann M, Clum A, Pillay M, Palaniappan K (2017). Genome data provides high support for generic boundaries in *Burkholderia* sensu lato. Front Microbiol.

[8] MACROGEN INC. [http://foreign.macrogen.com/eng/].

[9] Weisburg WG, Barns SM, Pelletier DA, Lane DJ (1991). 16S ribosomal DNA amplification for phylogenetic study. J Bacteriol.

[10] Edgar RC (2004). MUSCLE: multiple sequence alignment with high accuracy and high throughput. Nucleic Acid Res.

[11] Guindon S, Gascuel O (2003). A simple, fast, and accurate algorithm to estimate large phylogenies by maximum likelihood. Syst Biol.

[12] Yang Z (1996). Among-site rate variation and its impact on phylogenetic analyses. Trends Ecol Evol.

[13] Anisimova M, Gascuel O (2006). Approximate likelihood-ratio test for branches: a fast, accurate, and powerful alternative. Syst Biol.

[14] Tamura K, Peterson D, Peterson N, Stecher G, Nei M, Kumar S (2011). MEGA5: molecular evolutionary genetics analysis using maximum likelihood, evolutionary distance, and maximum parsimony methods. Mol Biol Evol.

[15] Eid J, Fehr A, Gray J, Luong K, Lyle J, Otto G, Peluso P, Rank D, Baybayan P, Bettman B (2009). Real-time DNA sequencing from single polymerase molecules. Science.

[16] JGI. Joint Genome Institute [http://www.jgi.doe.gov/].

[17] Chin C-S, Alexander DH, Marks P, Klammer AA, Drake J, Heiner C, Clum A, Copeland A, Huddleston J, Eichler EE (2013). Nonhybrid, finished microbial genome assemblies from long-read SMRT sequencing data. Nat Methods.

[18] Hyatt D, Chen G-L, LoCascio PF, Land ML, Larimer FW, Hauser LJ (2010). Prodigal: prokaryotic gene recognition and translation initiation site identification. BMC Bioinformatics.

[19] Pati AI, Mikhailova N, Ovchinikova N, Hooper G, Lykidis S, Kyrpides A, GenePRIMP N (2010). A gene prediction improvement pipeline for microbial genomes. Nat Methods.

[20] Lowe TM, Eddy SR (1997). tRNAscan-SE: a program for improved detection of transfer RNA genes in genomic sequence. Nucleic Acid Res.

[21] Markowitz VM, Chen IMA, Palaniappan K, Chu K, Szeto E, Pillay M, Ratner A, Huang J, Woyke T, Huntemann M, Anderson I, Billis K, Varghese N, Mavromatis K, Pati A, Ivanova NN, Kyrpides N. IMG 4 version of the integrated microbial genomes comparative analysis system. Nucleic Acid Res. 2013;42:D560–D567. 10.1093/nar/gkt963 PMC3965111 24165883

[22] Walker R, Watkin E, Tian R, Bräu L, O’Hara G, Goodwin L, Han J, Lobos E, Huntemann M, Pati A (2014). Genome sequence of the acid-tolerant *Burkholderia* sp. strain WSM2230 from Karijini National Park, Australia. Stand Genomic Sci.

[23] Walker R, Watkin E, Tian R, Bräu L, O’Hara G, Goodwin L, Han J, Reddy T, Huntemann M, Pati A (2014). Genome sequence of the acid-tolerant *Burkholderia* sp. strain WSM2232 from Karijini National Park, Australia. Stand Genomic Sci.

[24] Richter M, Rosselló-Móra R (2009). Shifting the genomic gold standard for the prokaryotic species definition. Proc Natl Acad Sci USA.

[25] Goris J, Konstantinidis KT, Klappenbach JA, Coenye T, Vandamme P, Tiedje JM (2007). DNA–DNA hybridization values and their relationship to whole-genome sequence similarities. Int J Syst Evol Microbiol.

[26] Field D, Garrity G, Gray T, Morrison N, Selengut J, Sterk P, Tatusova T, Thomson N, Allen MJ, Angiuoli SV (2008). The minimum information about a genome sequence (MIGS) specification. Nat Biotechnol.

[27] Woese CR, Kandler O, Wheelis ML (1990). Towards a natural system of organisms: proposal for the domains Archaea, bacteria, and Eucarya. Proc Natl Acad Sci USA.

[28] Garrity GM, Bell JA, Lilburn TE, Garrity GM, Brenner DJ, Krieg NR, Staley JT (2005). Class II. *Betaproteobacteria*. Bergey’s manual of systematic bacteriology.

[29] Garrity GM, Bell JA, Lilburn TE, Garrity GM, Brenner DJ, Krieg NR, Staley JT (2005). Class II. *Betaproteobacteria*. Bergey’s manual of systematic bacteriology.

[30] Garrity GM, Bell JA, Lilburn TE, Garrity GM, Brenner DJ, Krieg NR, Staley JT (2005). Order 1. *Burkholderiales*. Bergey’s manual of systematic bacteriology.

[31] Garrity GM, Bell JA, Lilburn TE, Family I, Garrity GM, Brenner DJ, Krieg NR, Staley JT (2005). Burkholderiaceae. Bergey’s manual of systematic bacteriology.

[32] Yabuuchi E, Kosako Y, Oyaizu H, Yano I, Hotta H, Hashimoto Y, Ezaki T, Arakawa M (1992). Proposal of *Burkholderia* gen. Nov. and transfer of seven species of the genus *Pseudomonas* homology group II to the new genus, with the type species *Burkholderia cepacia* (Palleroni and Holmes 1981) comb. nov. Microbiol Immunol.

[33] Ashburner M, Ball CA, Blake JA, Botstein D, Butler H, Cherry JM, Davis AP, Dolinski K, Dwight SS, Eppig JT (2000). Gene ontology: tool for the unification of biology. Nat Genet.

